# Efficiency of Early Sequential Laser Treatment for Facial Linear Scars in Cross‐Sectional Regions

**DOI:** 10.1111/jocd.70053

**Published:** 2025-02-13

**Authors:** Lei Guo, Ping Xue, Xing Fan, Yue Yin, Wenjie Dou, Tong Li, Qing Yang

**Affiliations:** ^1^ Department of Plastic and Reconstructive Surgery, Xijing Hospital Fourth Military Medical University Xi'an China

**Keywords:** carbon dioxide ablative fractional laser, long linear scar, pulsed dye laser, sequential treatment

## Abstract

**Background:**

Long linear scars in cross‐sectional regions can adversely affect facial aesthetics and functionality, leading to substantial psychological distress. Early intervention with carbon dioxide ablative fractional laser (CO_2_‐AFL) and 595‐nm pulsed dye laser (PDL) has shown promise in mitigating post‐surgical scarring. However, the effectiveness of this treatment for extensive facial scars across different areas remains unclear.

**Methods:**

We reviewed medical records of 39 patients with long scars from facial trauma between January 2022 and October 2023. Treatment commenced with two sessions of PDL and three sessions of CO_2_‐AFL 1 week post‐suture removal. Outcomes were assessed using Antera 3D imaging and the Patient and Observer Scar Assessment Scale (POSAS 3.0). Quality of life improvements were measured using the Short Form‐36 Health Survey (SF‐36).

**Results:**

All patients completed five sessions of laser treatment. Significant reductions were noted in Patient and Observer scores on the POSAS (*p* < 0.05). Antera 3D analysis revealed substantial improvements in average roughness, depression depth, and elevation depth across facial regions. The significance of improvements in color variation, texture elevation span, melanin hyperconcentration, and hemoglobin hyperconcentration varied by area. Patients also showed significant improvements in SF‐36 scores for physical role limitations, social functioning, and emotional well‐being compared to pre‐treatment levels (*p* < 0.05).

**Conclusions:**

Early sequential treatment with PDL and CO_2_‐AFL effectively improves long scars in various facial areas.

## 1 Introduction

Scarring is a normal result of skin tissue repair following trauma. When significant scars remain after facial injuries, they not only affect aesthetic appearance but also impose considerable physiological and psychological burdens on patients. Long scars resulting from trauma that cross multiple facial aesthetic units can severely impair the functional capabilities of facial tissues and organs, exacerbating the physiological and psychological trauma experienced by patients [1]. Therefore, early intervention in the skin healing process is crucial to prevent abnormal inflammatory responses, cell proliferation, and excessive collagen deposition that can lead to hypertrophic scarring, ultimately facilitating scar‐free healing of the injured tissue. Currently, methods for preventing scar hypertrophy include topical silicone preparations, intralesional corticosteroid injections, surgical excision, chemical peeling, pressure therapy, and radiation therapy [2]. In recent years, laser therapy has garnered increased attention due to its minimally invasive nature, low risk, and short recovery time [3, 4]. Early intervention with ablative carbon dioxide laser combined with 595‐nm pulsed dye laser has demonstrated promising results in improving scar texture and achieving scar‐free healing [5, 6]. However, the efficacy of combined laser treatments in enhancing the texture and pigmentation of early post‐traumatic scars in cross‐sectional facial areas, as well as the prognostic differences among various facial regions, remains uncertain.

## 2 Patients and Methods

**FIGURE 1:**
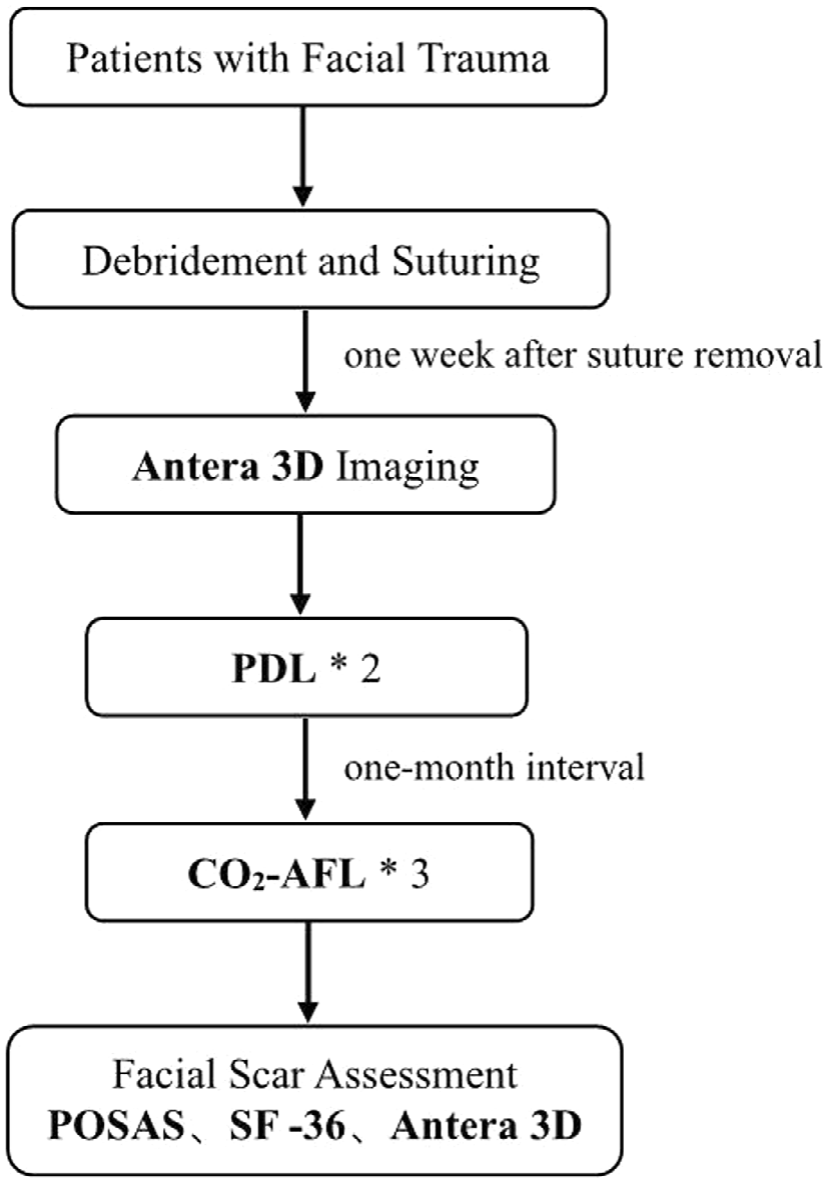
Flowchart for facial trauma scar treatment.

This study adheres to the Helsinki Declaration and has been approved by the ethics committee. All patients have provided informed consent. A total of 39 patients with long linear scars following facial trauma were included in this retrospective study, conducted between January 2022 and October 2023 (Figure 1). Inclusion criteria were as follows: outpatient status within 3 weeks post‐facial trauma suturing; age over 18 years; Fitzpatrick skin types III or IV; and facial scar length exceeding 5 cm(measured as the sum of the longest dimensions of linear scars), crossing multiple facial aesthetic units. Exclusion criteria included patients with systemic diseases such as chronic kidney disease, diabetes, and autoimmune disorders, as well as those with photosensitive skin conditions, and individuals deemed unsuitable for PDL or CO2‐AFL treatment were excluded.

### 2.1 Laser Treatment

All scars initially received treatment with a 595 nm variable pulse width pulsed dye laser (PDL) (V‐beam, Candela Corp., Wayland, MA, USA), using a spot size of 7 mm to ensure no overlap. The energy settings ranged from 7.0 to 7.5 J/cm^2^, with a pulse width of 0.45 ms. The endpoint for each PDL treatment was the occurrence of purpura in the treatment area, with sessions conducted every 4 weeks, totaling two cycles. Ice packs were applied post‐treatment to alleviate postoperative swelling and discomfort. A collagen dressing (Chuangfukang, Guangzhou Chuang'er Biotechnology Co. Ltd.,1500 IU/mL) was used for moisturizing within the week following treatment.

Upon completion of the two cycles of PDL treatment, patients underwent carbon dioxide ablative fractional laser (CO_2_‐AFL) treatment (Lumenis, Yokneam, Israel), set to the Deep FX mode, with a fluence range of 20.0–22.5 mJ/pulse, a density of 5%–10%, a frequency of 150 Hz, and a spot size of 120 μm. Each AFL treatment was spaced 8 weeks apart, with a total of three treatment cycles. Post‐treatment ice packs were also applied to reduce swelling and discomfort. During each treatment cycle, a topical application of recombinant bovine basic fibroblast growth factor gel (Zhuhai Yisheng Biopharmaceutical Co. Ltd., 42 000 IU/10 g) was administered 4–6 times daily for seven consecutive days to promote healing.

### 2.2 Linear Scar Assessment

We utilized an Antera 3D camera (Miravex, Dublin, Ireland) to capture scar images of patients at three time points: before treatment, 6 months after the first treatment, and 12 months after the first treatment. Objective evaluations of the scars included seven parameters: color variation (CV), texture average roughness (TAR), texture elevation span (TES), maximum depression depth (DMD), maximum elevation depth (EMD), melanin hyperconcentration (MH), and hemoglobin hyperconcentration (HH). These parameters assess various aspects of the scars, including color, texture, height, melanin, and hemoglobin levels [7]. The facial areas were divided into seven functional and aesthetic zones: forehead, temporal region, periorbital region, nasal region, malar and midface region, perioral region, and chin and jawline. Evaluations were conducted for each zone.

We employed the Patient and Observer Scar Assessment Scale (POSAS 3.0) linear version for subjective assessments of linear scars across the facial aesthetic zones [8, 9]. The Observer Scar Assessment Scale (OSAS) was scored by experienced cosmetic doctors who were not involved in the patients' treatments, while the Patient Scar Assessment Scale (PSAS) was completed by the patients themselves. All subjective assessments were conducted 12 months after the initial laser treatment.

### 2.3 Quality of Life Assessment

We employed the validated Short Form‐36 Health Survey (SF‐36) to assess patients' quality of life both before the first treatment and 12 months after the initial treatment.

### 2.4 Statistical Analysis

We performed data analysis using SPSS version 25.0 (IBM Statistics for Windows, IBM Corp., Armonk, NY, USA). All data were expressed as mean ± standard deviation or median (range). Antera 3D data were analyzed using One‐Way ANOVA, POSAS data were analyzed using paired sample *t*‐tests, and SF‐36 data were analyzed using the Wilcoxon signed‐rank test for differences. Statistical significance was set at *p* < 0.05.

## 3 Results

A total of 39 patients with linear scars in facial subunits were included in this study, consisting of 34 males and 5 females, with a median age of 36 years. In total, 84 scar sites across the facial subunits were involved (Table 1). All patients completed five sessions of laser treatment and adhered to post‐treatment care as instructed. Throughout all treatment cycles, no patients reported adverse reactions such as blister formation, hypertrophic scarring, or bacterial infection.

**TABLE 1:** Demographic data.

Characteristics	Patients with linear scar (*N* = 39)
Age, median(range)	36 (23–45)
Gender	34 M 5 F
Fitzpatrick skin type, *N* (%)	
III	15 (38.5%)
IV	24 (61.5%)
Scar length (cm), *N*(%)	
5–10	15 (38.5%)
10–15	19 (48.7%)
> 15	5 (12.8%)
Facial region, *N*(%)	
Forehead	13 (15.5%)
Temporal	9 (11.0%)
Periorbital	7 (8.3%)
Nasal	11 (13.1%)
Malar and midface	19 (22.6%)
Perioral	12 (14.3%)
Chin and jawline	13 (15.5%)

Abbreviations: F, female; M, male.

Among the Antera 3D measured parameters, melanin hyperconcentration (MH) did not show significant differences before and after treatment in the forehead, midface, and chin regions (*p* > 0.05). Hemoglobin hyperconcentration (HH) did not show significant changes in the periorbital and perioral regions (*p* > 0.05). Color variation (CV) showed no significant difference in the periorbital region (*p* > 0.05), and texture average roughness (TAR) showed no significant change in the perioral region (*p* > 0.05). Significant differences were observed in texture elevation span (TES), depression maximum depth (DMD), and elevation maximum depth (EMD) across all facial subunits (*p* < 0.05).

Regionally, significant differences were noted in the temporal and nasal regions across all parameters, followed by the forehead, midface, and chin regions, where only MH showed no significant changes. The periorbital and perioral regions showed a smaller number of parameters with significant differences (Table 2, Table S1). Regarding the degree of improvement, the forehead, midface, and nasal regions showed improvements of over 50% in most parameters 12 months post‐treatment, while the periorbital and perioral regions showed relatively smaller improvements.

**TABLE 2:** Antera 3D data.

Facial segment	T0	T1	T2	(T0−T1)/T0 (%)	(T0−T2)/T0 (%)	*p*		
						T0 versus T1	T0 versus T2	T1 versus T2
Forehead								
CV	3.886 ± 0.660	3.342 ± 0.546	2.952 ± 0.403	13.999	24.035	0.016^a^	0.000^a^	0.078
TAR	17.394 ± 7.685	9.036 ± 1.325	7.985 ± 1.083	48.051	54.093	0.000^a^	0.000^a^	0.559
TES	0.444 ± 0.236	0.250 ± 0.162	0.095 ± 0.016	43.694	78.604	0.005^a^	0.000^a^	0.023
DMD	0.290 ± 0.175	0.131 ± 0.059	0.069 ± 0.026	54.828	76.207	0.001^a^	0.000^a^	0.143
EMD	0.274 ± 0.150	0.084 ± 0.035	0.056 ± 0.012	69.343	79.562	0.000^a^	0.000^a^	0.423
MH	34.216 ± 17.717	28.642 ± 15.980	27.830 ± 15.628	16.291	18.664	0.394	0.329	0.901
HH	32.483 ± 23.895	13.415 ± 13.069	7.834 ± 8.336	58.701	75.883	0.005^a^	0.001^a^	0.393
Periorbital								
CV	4.322 ± 1.228	3.527 ± 0.641	3.436 ± 0.865	18.404	20.492	0.132	0.096	0.860
TAR	17.680 ± 2.880	12.708 ± 4.338	11.538 ± 4.047	28.125	34.740	0.025^a^	0.007^a^	0.573
TES	0.194 ± 0.071	0.148 ± 0.054	0.127 ± 0.039	23.819	34.366	0.142	0.040^a^	0.505
DMD	0.139 ± 0.030	0.088 ± 0.039	0.080 ± 0.036	36.722	42.566	0.014^a^	0.005^a^	0.668
EMD	0.124 ± 0.056	0.119 ± 0.043	0.074 ± 0.027	4.364	40.529	0.820	0.046^a^	0.071
MH	24.564 ± 7.244	18.466 ± 4.919	14.780 ± 3.515	24.823	38.829	0.051	0.003^a^	0.222
HH	17.778 ± 19.094	13.796 ± 11.812	7.512 ± 7.362	22.400	57.746	0.235	0.176	0.175
Midface								
CV	6.709 ± 1.909	3.622 ± 0.602	3.091 ± 0.408	46.013	53.928	0.000^a^	0.000^a^	0.171
TAR	14.212 ± 7.522	10.196 ± 2.876	6.003 ± 1.219	28.258	57.761	0.011^a^	0.000^a^	0.008
TES	0.227 ± 0.121	0.114 ± 0.036	0.083 ± 0.027	49.780	63.463	0.000^a^	0.000^a^	0.217
DMD	0.139 ± 0.082	0.091 ± 0.028	0.055 ± 0.021	34.532	60.432	0.005^a^	0.000^a^	0.037^a^
EMD	0.190 ± 0.100	0.155 ± 0.208	0.046 ± 0.013	18.421	75.789	0.142	0.002^a^	0.032^a^
MH	21.764 ± 14.995	21.369 ± 13.936	20.329 ± 12.272	1.815	6.593	0.930	0.750	0.817
HH	57.011 ± 35.269	26.313 ± 17.489	11.564 ± 8.831	53.846	79.716	0.000^a^	0.000^a^	0.056

Abbreviations: CV, color variation; DMD, depression max depth; EMD, elevation max depth; HH, haemohlobin hyperconcentration; MH, melanin hyperconcentration; T0, Pre‐treatment; T1, 6 months post‐treatment; T2, 12 months post‐treatment; TAR, texture average roughness; TES, texture elevation span.

^a^ Indicates statistical significance.

In the POSAS, patient‐reported items for pain, fragility, and widening showed low scores both before and after treatment, with no significant differences (*p* > 0.05). For observer‐reported items, only the widened parameter showed no significant difference (*p* > 0.05), while all other scores demonstrated significant changes (Table 3, Tables S2 and S3). SF‐36 analysis revealed significant improvements in the limitations in physical role, social functioning, and emotional well‐being scores post‐treatment (*p* < 0.05), while no significant differences were observed in other quality of life parameters (Table 4).

**TABLE 3:** Patient and observer scar assessment scale.

Variables	Pre‐treatment	Post‐treatment	*p*
Patient			
Overall quality	4.51 ± 0.51	1.77 ± 0.84	0.00^a^
Pain	2.36 ± 1.16	1.97 ± 0.81	0.09
Itch	4.10 ± 0.78	1.82 ± 0.79	0.00^a^
Color	2.87 ± 0.80	2.08 ± 0.87	0.00^a^
Height	3.92 ± 0.77	2.00 ± 0.76	0.00^a^
Hardness	2.79 ± 0.83	2.03 ± 0.74	0.00^a^
Fragility	1.95 ± 0.79	1.56 ± 0.50	0.13
Widening	2.00 ± 0.80	2.33 ± 0.81	0.07
Irregularity	4.00 ± 0.76	1.92 ± 0.84	0.00^a^
PSAS total score	58.31 ± 4.38	34.95 ± 3.49	0.00^a^
Observer			
Overall quality	4.05 ± 0.89	1.82 ± 0.82	0.00^a^
Pigmentation	3.00 ± 0.83	2.13 ± 0.83	0.00^a^
Vascularity	2.92 ± 0.74	1.97 ± 0.81	0.00^a^
Surface texture	3.95 ± 0.79	1.46 ± 0.51	0.00^a^
Firm	2.85 ± 0.75	1.49 ± 0.51	0.00^a^
Adhered	4.05 ± 0.79	1.82 ± 0.82	0.00^a^
Widened	2.08 ± 0.84	1.41 ± 0.50	0.89
OSAS total score	33.92 ± 2.83	17.08 ± 2.58	0.00^a^

*Note:* All data are presented as mean ± standard deviation and analyzed using the *t* test.

^a^ Indicates statistical significance.

**TABLE 4:** Short form‐36.

Variables	Pre‐treatment	Post‐treatment	*p*
Physical functioning	75 (25–100)	75 (50–100)	0.49
Limitations' role physical	50 (50–100)	75 (50–100)	0.01^a^
Bodily pain	80 (60–100)	90 (60–100)	0.13
General health perception	90 (75–100)	90 (75–100)	0.36
Vitality	85 (70–100)	85 (70–100)	0.74
Social functioning	33 (11–67)	78 (44–100)	0.00^a^
Limitations' role emotional	33 (0–67)	33 (0–67)	0.44
Emotional well‐being	44 (20–60)	70 (52–100)	0.00^a^

*Note:* All data are presented as median (range) and analyzed using the Wilcoxon signed‐rank test.

^a^ Indicates statistical significance.

## 4 Cases Report

### 4.1 Case 1

**FIGURE 2:**
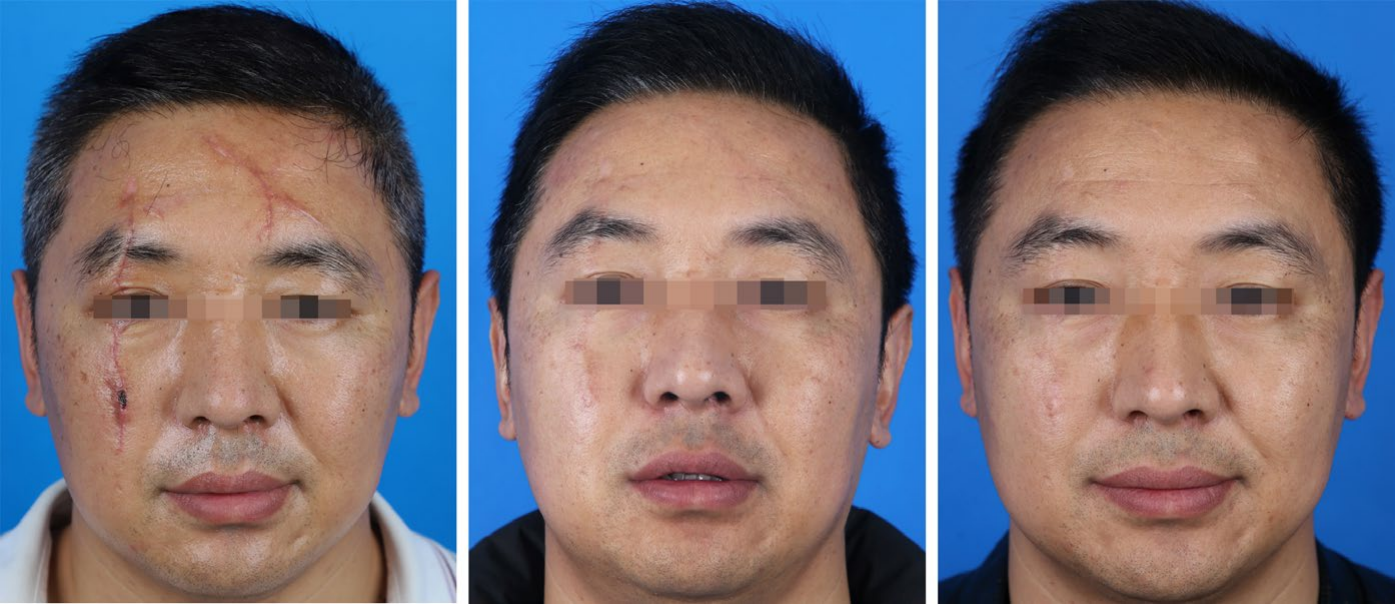
A 38‐year‐old male with linear scars on the face affecting the forehead, periorbital region, and midface, with a cumulative length of approximately 15 cm. The images show the scars before treatment, 6 months post‐initial treatment, and 12 months post‐initial treatment.

**FIGURE 3:**
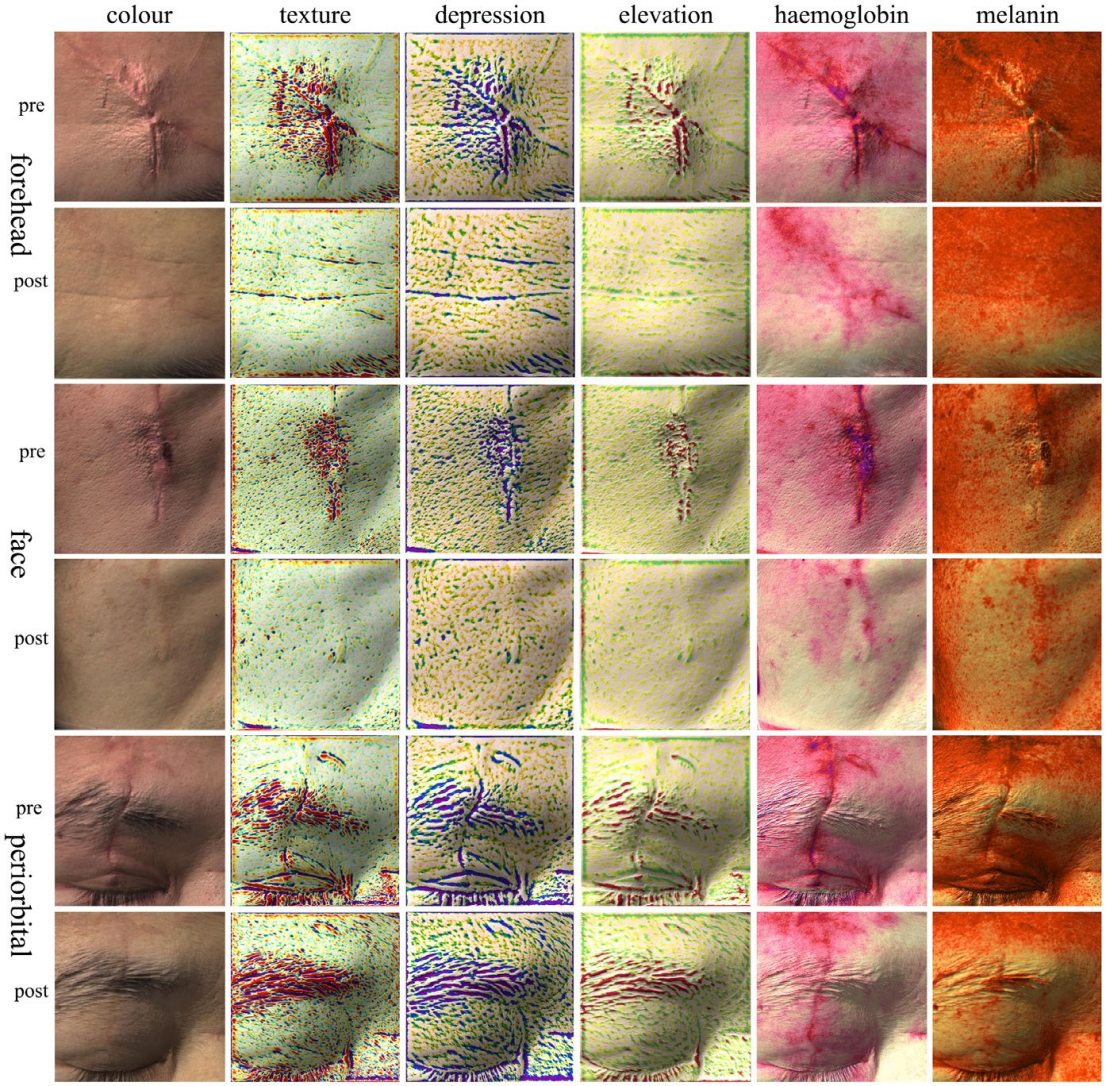
Antera 3D images of a 38‐year‐old male showing the treatment area of scars in three facial regions pre‐treatment and 12 months post‐initial treatment. There is a significant improvement in the texture, height, and color of the scars across all three regions. Notably, hemoglobin accumulation remains evident in the periorbital region 12 months after initial treatment. In the midface area, melanin accumulation is slightly more pronounced than in the surrounding normal tissue. Hemoglobin accumulation is still significant in the diagonal scar of the forehead region post‐treatment.

A 38‐year‐old male presented with linear facial scars affecting the forehead, periorbital region, and midface following suturing for facial trauma over 2 weeks prior. The total length of the scars was 15 cm, significantly impacting his facial appearance and perceived social interactions, prompting him to seek scar management treatment at a cosmetic clinic. The patient began sequential treatments with PDL and CO2‐AFL 1 week after suture removal, completing a total of five laser treatment cycles. There was a marked improvement in scar texture and appearance post‐treatment, with PSAS and OSAS of 32 and 15, respectively. The patient expressed high satisfaction with the final treatment outcomes, noting that the scars had minimal impact on the functional and aesthetic aspects of facial features and reported a significant improvement in psychological well‐being (Figures 2 and 3).

### 4.2 Case 2

**FIGURE 4:**
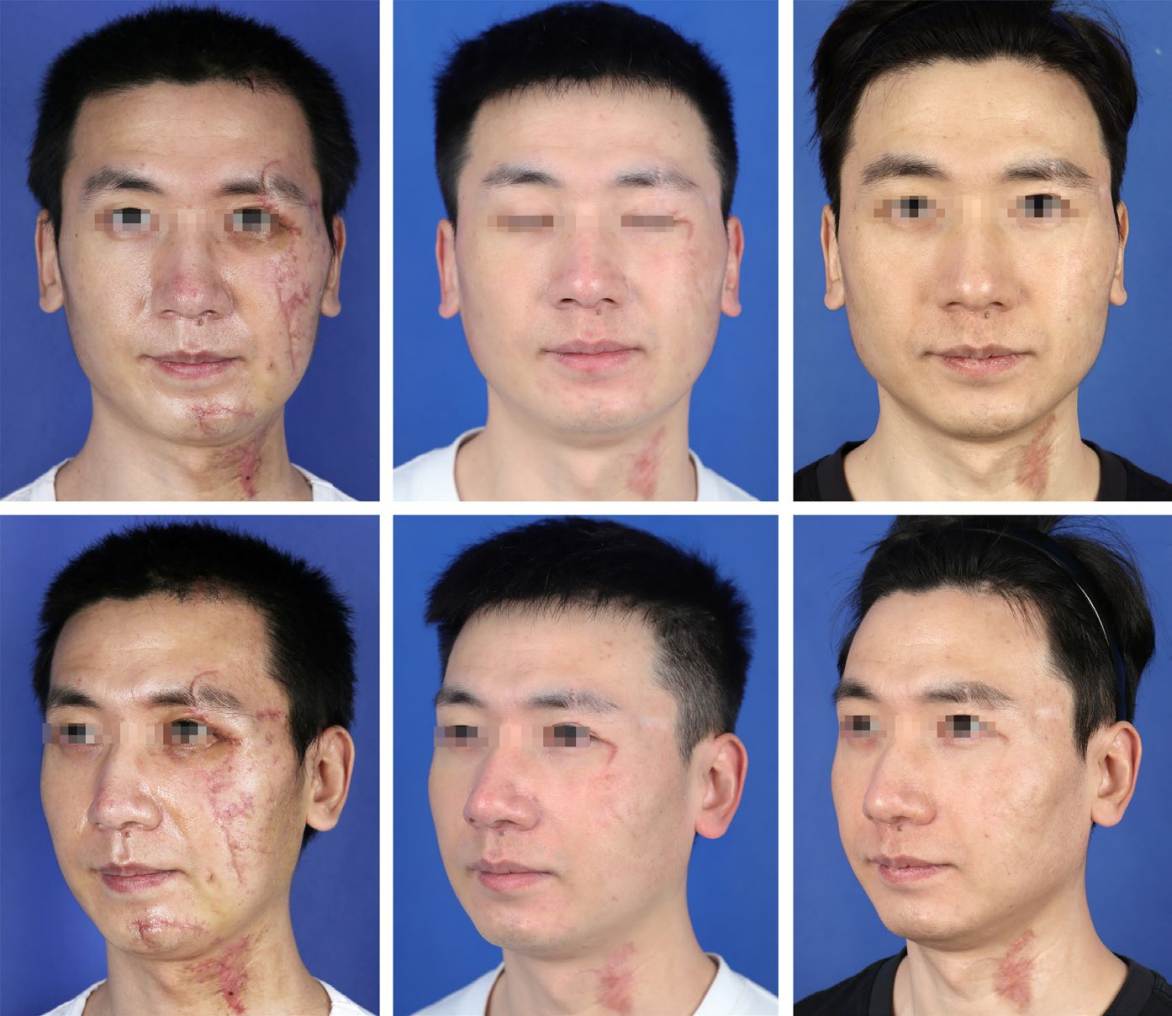
A 32‐year‐old male with linear scars affecting five regions of the face: Periorbital region, midface, chin, temporal region, and neck, with a total length of 27 cm. The images depict the scars before treatment, 6 months post‐initial treatment, and 12 months post‐initial treatment (the neck scar did not receive laser treatment).

**FIGURE 5:**
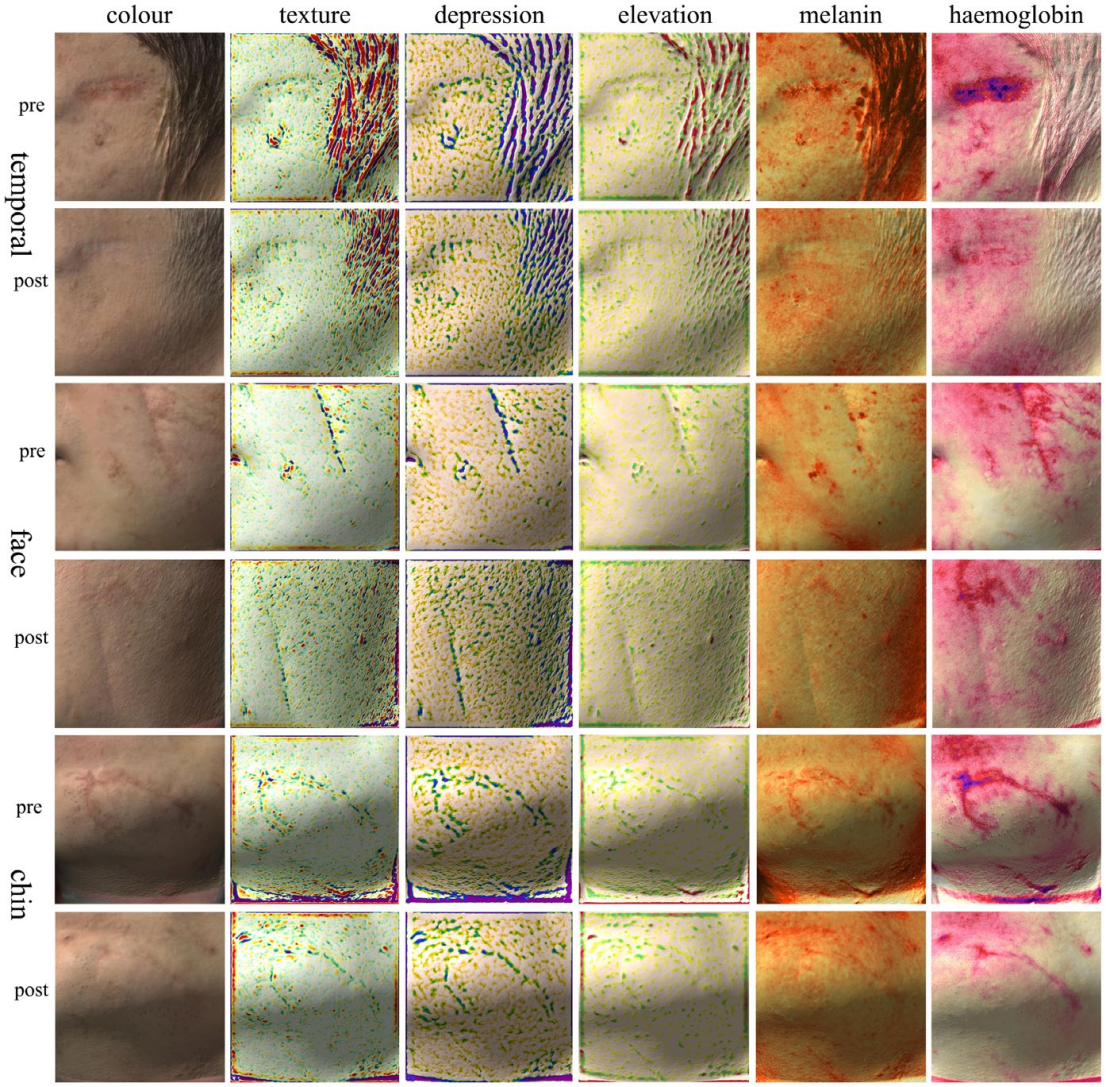
Antera 3D images of a 32‐year‐old male showing the treatment area of scars in three facial regions before treatment and 12 months post‐initial treatment. There is a significant improvement in the texture, height, and color of the scars in all three regions. In particular, the scar near the perioral region shows notable hemoglobin accumulation 12 months post‐initial treatment, along with the presence of shallow grooves.

A 32‐year‐old male presented 3 weeks after suturing for facial trauma, with linear scars affecting the periorbital region, midface, chin, temporal region, and neck, totaling 27 cm in length. The scars significantly impacted the patient's facial appearance and caused psychological distress, leading him to seek scar management treatment at a cosmetic clinic. The patient initiated sequential treatments with PDL and CO2‐AFL 2 weeks after suture removal, completing a total of five laser treatment cycles, resulting in significant improvements in scar texture and appearance. The scars on the neck did not receive laser intervention and, after 12 months, exhibited contraction and persistent erythematous texture. Post‐treatment scores on the PSAS and OSAS were 35 and 17, respectively. The patient expressed high satisfaction with the final treatment outcomes and reported a noticeable improvement in psychological well‐being (Figures 4 and 5). However, the scar in the lower facial region near the mouth showed adhesion to the deeper tissues, resulting in a prominent groove during contractions of the facial expression muscles (Video S1).

## 5 Discussion

Scars refer to permanent skin damage that occurs when skin injuries extend to the reticular layer of the dermis. This condition results from an imbalance in collagen synthesis and degradation during the inflammatory response and tissue repair process, leading to excessive proliferation of fibrous connective tissue [10]. Facial scars are particularly conspicuous and difficult to conceal, adversely affecting aesthetic appearance. They may also cause discomfort, including pain, hardness, and itching, and can lead to functional issues such as difficulties with eating, speaking, nasal breathing, and eyelid closure. More importantly, these scars impose a lasting and significant psychological burden on patients, potentially influencing how individuals are perceived by society and leading to misunderstandings or biases against scarred individuals, ultimately impacting their social interactions and professional development [11, 12].

Following tissue injury, wound healing progresses through three interconnected phases: the inflammatory phase (days 1–3), the proliferative phase (days 4–21), and the remodeling phase. During the inflammatory phase, vasodilation occurs, increasing blood flow to the area, which facilitates the removal of pathogens and necrotic tissue through the inflammatory response. In the proliferative phase, fibroblasts generate collagen and elastin fibers, forming a matrix scaffold in the wound area. The remodeling phase involves the rearrangement of collagen at the wound site, with a reduction in angiogenesis leading to the gradual stabilization of a regular vascular network. Normal wound healing and scar remodeling typically last for 2 to 3 years. Patients with facial scars often seek professional scar management treatments for faster repair and improved aesthetics [10, 13].

Laser treatments are effective in restoring the natural, even color of scars and reducing irregular contours, making them a preferred option for patients with facial scars. In 1983, Anderson and Parisy [14] introduced the principle of selective photothermolysis, which provided a significant theoretical foundation for laser therapy. In 1994, Alster [15] reported the use of PDL for the treatment of hypertrophic scars, observing a marked improvement in the clinical symptoms of the scars. PDL reduces nutrient delivery to the scar by selectively damaging microvessels, obstructing the abnormal dilation of capillaries in the scar, and disrupting the microenvironment that facilitates oxygen transport and nutrient supply. CO_2_ lasers remove scars through the processes of burning, vaporization, or carbonization. In 2004, Manstein [16] proposed the principle of fractional photothermolysis, leading to a significant increase in the popularity of laser treatment for scars. The widely used CO_2_ fractional laser applies a matrix of laser beams to the scar or skin tissue, allowing the water in the tissue to absorb laser energy and vaporize, creating multiple three‐dimensional columnar microthermal treatment zones (MTZ). This method preserves the undamaged skin tissue between the MTZ, facilitating healing after laser treatment while directly vaporizing scar tissue, activating the tissue's self‐repair mechanisms, and improving collagen structure. Cho and Makboul [17, 18] successfully employed CO_2_‐AFL to treat hypertrophic scars, achieving notable therapeutic results. Recent studies have emphasized the advantages of sequential treatment with PDL and CO_2_‐AFL, demonstrating an additive effect that surpasses the efficacy of either treatment alone. Ouyang HW [19] has reported successful outcomes in the combined treatment of immature red hypertrophic scars, while Liu XJ [20] achieved favorable results in treating hypertrophic scars in children following burns. Kivi MK [21] also noted excellent therapeutic results with the combined use of two lasers for burn‐related hypertrophic scars. Additionally, numerous studies have explored the timing of laser interventions, suggesting that early laser treatment, typically within 1 week after suture removal, can yield better outcomes [5, 6, 21]. Most prior research on the combination of two lasers has focused on hypertrophic scars or keloids. Currently, there are no reported studies concerning the use of two lasers for scar prevention following traumatic suturing, particularly for linear facial scars.

Facial scars, characterized by linear formations that traverse multiple aesthetic zones and involve various functional units, present significant treatment challenges, particularly for longer scars, which may result in inferior long‐term outcomes. We observed that the early application of sequential PDL and CO_2_‐AFL treatments within 2 to 3 weeks post‐surgery can significantly enhance the appearance, texture, and pigmentation of scars, thereby facilitating the remodeling process and promoting early scar‐free healing. Comparative analysis of pre‐ and post‐treatment Antera 3D images revealed that sequential laser treatment significantly improves scar texture, alleviating both elevations and depressions while reducing the overall roughness of the scars. However, in terms of reducing hemoglobin accumulation, the improvement in dynamic facial areas was slightly less effective than in static regions. This may be attributed to the persistent muscular activity in deeper scar tissue, which increases blood flow around the wound, particularly evident in the periorbital region. In terms of melanin accumulation, laser treatment showed minimal improvement in the forehead, midface, and chin areas, potentially due to the susceptibility of these regions to sun‐induced pigmentation.

Antera 3D imaging effectively visualizes changes before and after scar treatment, providing a precise depiction of subtle alterations that may not be perceptible to the naked eye, independent of observer experience or patient perception [5, 7]. However, we also found that while Antera 3D imaging is an accurate objective assessment tool for static scar conditions, it does not capture the dynamic state of scars. For instance, in Case 2, the static images depicted minimal scarring, but dynamic expressions revealed prominent grooves. Therefore, scar assessment should combine Antera 3D imaging with scar evaluation scales for a comprehensive evaluation.

Our study indicates significant differences in healing outcomes for linear scars across various facial regions, particularly in areas of frequent muscular activity, such as the periorbital, perioral, and lower facial regions, where the likelihood of developing grooves post‐treatment is notably higher and hemoglobin accumulation remains relatively elevated. We posit that this is more related to ongoing muscular activity rather than the laser treatment itself. Consequently, for scars in these active muscular areas, adjunctive therapies—such as botulinum toxin injections [22], pressure therapy, and surgical release combined with fat grafting [2, 23]—should be considered to achieve optimal treatment outcomes.

The JAMA consensus recommends selecting appropriate lasers for treatment based on scar characteristics, including skin color (erythema, hyperpigmentation, hypopigmentation), scar type (hypertrophic, flat, atrophic), location (face, neck, limbs), and patient characteristics (skin type and comorbid conditions) [24]. During the treatment with a 595 nm wavelength pulsed dye laser (PDL), melanin acts as a competitive chromophore to hemoglobin [25]. At this wavelength, the absorption of epidermal melanin is relatively pronounced compared to that of oxyhemoglobin, particularly in individuals with higher Fitzpatrick skin types [26]; therefore, energy settings should be adjusted according to skin color. Specifically, for individuals with darker skin tones, the energy settings should be relatively lower. Additionally, PDL treatment is not recommended for individuals who have been sun‐exposed in the past month, as excessive energy absorption in this population can lead to adverse effects such as blistering.

Our study used the updated POSAS 3.0 for subjective evaluation of long facial linear scars, Antera 3D imaging for objective assessment, and the SF‐36 scale for quality of life evaluation. Additionally, we conducted a comprehensive assessment of the early laser treatment effects on scars across facial regions. This study provides a reference for facial trauma scar management; however, it is important to note that our research still has several limitations. Given the differences in scar thickness and the variety of CO_2_‐AFL devices, the parameters we recommend are provided for reference only. Future research should explore the effects of different devices and treatment modes to establish evidence‐based technical guidelines. Tension is a significant factor influencing scar formation; in this study, the orientation of linear scars relative to the Relaxed Skin Tension Lines (RSTL) may also affect the final treatment outcomes, yet we did not differentiate this aspect, limiting the horizontal comparisons across facial regions [27]. We employed the POSAS to evaluate the overall condition of facial scars, but differences in patients' cognitive abilities and adherence prevented us from assessing scars in specific regions. Additionally, this study was a single‐center retrospective analysis with a relatively small sample size. Future research should include multi‐center randomized controlled trials to better evaluate treatment efficacy.

## 6 Conclusion

Early sequential treatment with PDL and CO_2_‐AFL demonstrates significant effectiveness in improving long linear scars across various facial regions, substantially enhancing patients' quality of life.

## Author Contributions

G.L., X.P., and D.W. performed the research. F.X. and Y.Q. supervised the research study. Y.Y. and L.T. analyzed the data. G.L. and X.P. wrote the paper.

## Conflicts of Interest

The authors declare no conflicts of interest.

## Supporting information


**Video S1.** Supplementary Video.

**Data S1.** Supplementary Tables.

**Video S2.** Video Legend.

## Data Availability

The data that support the findings of this study are available from the corresponding author upon reasonable request.
