# Supplementary material for: Efficiency of Early Sequential Laser Treatment for Facial Linear Scars in Cross‐Sectional Regions
Source: J Cosmet Dermatol. 2025 Feb 13;24(2):e70053. doi: 10.1111/jocd.70053 (PMC11822557; doi:10.1111/jocd.70053)
Supplement: Supplementary file 2 — Data S1. Supplementary Tables. [file JOCD-24-e70053-s001.docx]

**Supplementary Table.1** Antera 3D data

| Facial segment | T0 | T1 | T2 | （T0-T1）/ T0（%） | （T0-T2）/ T0（%） | *P* value | | |
| --- | --- | --- | --- | --- | --- | --- | --- | --- |
|  |  |  |  |  |  | T0 *VS* T1 | T0 *VS* T2 | T1 *VS* T2 |
| Perioral |  |  |  |  |  |  |  |  |
| CV | 4.134±0.563 | 3.619±0.438 | 2.987±0.486 | 12.486 | 27.753 | 0.016* | 0.000* | 0.004* |
| TAR | 14.004±2.748 | 12.081±1.987 | 9.612±2.399 | 13.733 | 31.358 | 0.058 | 0.000* | 0.017* |
| TES | 0.159±0.016 | 0.149±0.039 | 0.135±0.035 | 6.142 | 15.118 | 0.456 | 0.073 | 0.279 |
| DMD | 0.143±0.023 | 0.124±0.041 | 0.086±0.040 | 13.588 | 39.945 | 0.191 | 0.000* | 0.014* |
| EMD | 0.115±0.015 | 0.094±0.017 | 0.086±0.020 | 18.781 | 25.284 | 0.004* | 0.000* | 0.291 |
| MH | 14.109±3.518 | 11.159±5.080 | 10.455±4.367 | 20.911 | 25.901 | 0.108 | 0.048* | 0.696 |
| HH | 11.608±10.315 | 9.073±9.976 | 5.769±5.037 | 21.844 | 50.303 | 0.484 | 0.113 | 0.363 |
| Temporal |  |  |  |  |  |  |  |  |
| CV | 6.204±0.644 | 4.610±0.596 | 3.837±0.244 | 25.691 | 38.158 | 0.000* | 0.000* | 0.005* |
| TAR | 16.093±5.202 | 11.748±1.928 | 10.580±1.215 | 26.999 | 34.258 | 0.010* | 0.002* | 0.457 |
| TES | 0.193±0.047 | 0.130±0.033 | 0.085±0.224 | 32.530 | 40.516 | 0.327 | 0.020* | 0.002* |
| DMD | 0.132±0.033 | 0.079±0.027 | 0.076±0.017 | 39.834 | 41.946 | 0.000* | 0.000* | 0.824 |
| EMD | 0.100±0.023 | 0.074±0.018 | 0.069±0.013 | 25.833 | 30.712 | 0.006* | 0.002* | 0.577 |
| MH | 25.192±1.719 | 22.024±1.823 | 19.484±1.769 | 12.575 | 22.656 | 0.001* | 0.000* | 0.006* |
| HH | 71.156±22.123 | 23.403±1.229 | 12.879±2.538 | 67.110 | 81.900 | 0.000* | 0.000* | 0.096 |
| Nasal |  |  |  |  |  |  |  |  |
| CV | 5.472±1.020 | 5.312±1.191 | 4.238±0.871 | 2.924 | 22.556 | 0.732 | 0.013* | 0.028* |
| TAR | 17.963±6.141 | 12.345±2.774 | 9.862±1.833 | 31.274 | 45.100 | 0.004* | 0.000* | 0.180 |
| TES | 0.225±0.098 | 0.153±0.053 | 0.111±0.023 | 31.982 | 50.646 | 0.021* | 0.001* | 0.165 |
| DMD | 0.214±0.080 | 0.138±0.055 | 0.080±0.024 | 35.544 | 62.868 | 0.006* | 0.000* | 0.031* |
| EMD | 0.160±0.043 | 0.121±0.028 | 0.083±0.227 | 24.735 | 48.032 | 0.515 | 0.024* | 0.005* |
| MH | 28.247±5.852 | 22.412±4.351 | 16.994±4.016 | 20.656 | 39.835 | 0.011* | 0.000* | 0.018* |
| HH | 27.038±13.589 | 16.718±8.160 | 7.814±3.385 | 38.170 | 71.099 | 0.020* | 0.000* | 0.043* |
| Chin |  |  |  |  |  |  |  |  |
| CV | 5.115±0.194 | 3.594±0.449 | 3.319±0.333 | 29.748 | 35.109 | 0.000* | 0.000* | 0.048* |
| TAR | 14.861±4.044 | 10.284±1.223 | 8.817±0.689 | 30.797 | 40.666 | 0.000* | 0.000* | 0.139 |
| TES | 0.479±0.330 | 0.125±0.010 | 0.102±0.006 | 73.852 | 78.812 | 0.000* | 0.000* | 0.752 |
| DMD | 0.130±0.028 | 0.075±0.006 | 0.076±0.011 | 42.635 | 41.282 | 0.000* | 0.000* | 0.797 |
| EMD | 0.102±0.012 | 0.068±0.005 | 0.066±0.011 | 32.961 | 35.147 | 0.000* | 0.000* | 0.572 |
| MH | 23.061±4.441 | 20.593±5.098 | 23.442±4.972 | 10.701 | -1.655 | 0.202 | 0.842 | 0.143 |
| HH | 40.111±17.585 | 13.443±5.954 | 5.999±1.393 | 66.485 | 85.045 | 0.000* | 0.000* | 0.086 |

CV:colour variation TAR:texture average roughness TES:texture elevation span DMD:depression max depth EMD:elevation max depth MH:melanin hyperconcentration HH:haemohlobin hyperconcentration

T0：Pre-treatment T1：6 months post-treatment T2：12 months post-treatment

* indic

**Supplementary Table.2** OSAS scale

| Variables | Pre-treatment | Post-treatment | P value |
| --- | --- | --- | --- |
| overall quality | 4.05±0.89 | 1.82±0.82 | 0.00* |
| pigmentation | 3.00±0.83 | 2.13±0.83 | 0.00* |
| vascularity | 2.92±0.74 | 1.97±0.81 | 0.00* |
| surface level | 4.00±0.89 | 2.03±0.74 | 0.00* |
| surface texture | 3.95±0.79 | 1.46±0.51 | 0.00* |
| firm | 2.85±0.75 | 1.49±0.51 | 0.00* |
| adhered | 4.05±0.79 | 1.82±0.82 | 0.00* |
| tension | 3.05±0.72 | 1.44±0.50 | 0.00* |
| widened | 2.08±0.84 | 1.41±0.50 | 0.89 |
| marks of surgical  wound closure | 3.97±0.74 | 1.51±0.51 | 0.00* |
| OSAS total score | 33.92±2.83 | 17.08±2.58 | 0.00* |

All data are presented as mean±standard deviation and analyzed using the t test;

* indicates statistical significance.

**Supplementary Table.3** PSAS scale

| Variables | Pre-treatment | Post-treatment | P value |
| --- | --- | --- | --- |
| overall quality | 4.51±0.51 | 1.77±0.84 | 0.00* |
| color | 2.87±0.80 | 2.08±0.87 | 0.00* |
| shine | 4.26±0.88 | 1.97±0.74 | 0.00* |
| height | 3.92±0.77 | 2.00±0.76 | 0.00* |
| hardness | 2.79±0.83 | 2.03±0.74 | 0.00* |
| irregularity | 4.00±0.76 | 1.92±0.84 | 0.00* |
| widening | 2.00±0.80 | 2.33±0.81 | 0.07 |
| sensitivity | 3.05±0.79 | 2.18±0.72 | 0.00* |
| numbness-insensitivity | 3.05±0.79 | 2.08±0.87 | 0.00* |
| pain | 2.36±1.16 | 1.97±0.81 | 0.09 |
| a shooting sensation | 4.00±0.83 | 2.03±0.90 | 0.00* |
| a burning sensation | 2.44±1.17 | 1.44±0.50 | 0.00* |
| itch | 4.10±0.78 | 1.82±0.79 | 0.00* |
| tingling and  pins and needles | 4.08±0.81 | 1.90±0.82 | 0.00* |
| tighness | 2.97±0.81 | 2.05±0.86 | 0.00* |
| pilling tight  with movement | 3.85±0.71 | 2.31±1.10 | 0.00* |
| fragility | 1.95±0.79 | 1.56±0.50 | 0.13 |
| dryness | 2.10±0.82 | 1.51±0.51 | 0.00* |
| PSAS total score | 58.31±4.38 | 34.95±3.49 | 0.00* |

All data are presented as mean±standard deviation and analyzed using the t test;

* indicates statistical significance.
